# Exosomal MicroRNAs in Alzheimer’s Disease: Unveiling Their Role and Pioneering Tools for Diagnosis and Treatment

**DOI:** 10.3390/jcm13226960

**Published:** 2024-11-19

**Authors:** Alhanof Alhenaky, Safiah Alhazmi, Sultan H. Alamri, Heba A. Alkhatabi, Amani Alharthi, Mansour A. Alsaleem, Sameh A. Abdelnour, Sabah M. Hassan

**Affiliations:** 1 Department of Biological Sciences, Faculty of Science, King Abdulaziz University, Jeddah 21589, Saudi Arabia; 2 Immunology Unit, King Fahd Medical Research Center, King Abdulaziz University, Jeddah 80200, Saudi Arabia; 3 Neuroscience and Geroscience Research Unit, King Fahd Medical Research Center, King Abdulaziz University, Jeddah 80200, Saudi Arabia; 4 Department of Family Medicine, Faculty of Medicine, King Abdulaziz University, Jeddah 21589, Saudi Arabia; 5 Department of Medical Laboratory Technology, Faculty of Applied Medical Sciences, King Abdulaziz University, Jeddah 22254, Saudi Arabia; 6 Hematology Research Unit (HRU), King Fahd Medical Research Center, King Abdulaziz University, Jeddah 22254, Saudi Arabia; 7 Department of Biology, College of Science Al-Zulfi, Majmaah University, Majmaah 11952, Saudi Arabia; 8 Unit of Scientific Research, Applied College, Qassim University, Buraydah 52571, Saudi Arabia; 9 Department of Animal Production, Faculty of Agriculture, Zagazig University, Zagazig 44519, Egypt; 10 Princess Najla Bint Saud Al-Saud Center for Excellence Research in Biotechnology, King Abdulaziz University, Jeddah 21589, Saudi Arabia; 11 Department of Genetics, Faculty of Agriculture, Ain Shams University, Cairo 11517, Egypt

**Keywords:** Alzheimer’s disease, exosome, extracellular vesicles, microRNAs, treatment, biomarkers

## Abstract

Alzheimer’s disease (AD) is a common neurodegenerative disorder that presents a significant health concern, often leading to substantial cognitive decline among older adults. A prominent feature of AD is progressive dementia, which eventually disrupts daily functioning and the ability to live independently. A major challenge in addressing AD is its prolonged pre-symptomatic phase, which makes early detection difficult. Moreover, the disease’s complexity and the inefficiency of current diagnostic methods impede the development of targeted therapies. Therefore, there is an urgent need to enhance diagnostic methodologies for detection and treating AD even before clinical symptoms appear. Exosomes are nanoscale biovesicles secreted by cells, including nerve cells, into biofluids. These exosomes play essential roles in the central nervous system (CNS) by facilitating neuronal communication and thus influencing major physiological and pathological processes. Exosomal cargo, particularly microRNAs (miRNAs), are critical mediators in this cellular communication, and their dysregulation affects various pathological pathways related to neurodegenerative diseases, including AD. This review discusses the significant roles of exosomal miRNAs in the pathological mechanisms related to AD, focusing on the promising use of exosomal miRNAs as diagnostic biomarkers and targeted therapeutic interventions for this devastating disease.

## 1. Introduction

Alzheimer’s disease (AD) is an irreversible neurodegenerative disease marked by a gradual impairment of functioning and cognitive abilities, ultimately resulting in dementia. More than 55 million patients have dementia worldwide; AD accounts for 60–70% of all cases. It is estimated that the cases of AD could increase as the population ages, reaching 78 million by 2030 and 139 million by 2050 [1]. AD and other dementia cause a significant economic burden, with current healthcare costs estimated to be USD 355 billion in 2020 and possibly reach USD 1.1 trillion as the population ages [2]. In addition to costs, AD presents a challenge to healthcare due to its long pre-symptomatic phase, marked by the buildup of abnormal proteins and neurotoxicity in the brain [3,4], making the prediction or early detection of the disease extremely difficult. The inefficiency of current diagnostic methods and the disease’s complexity pose major obstacles to AD treatment. Consequently, there is an urgent need to research in order to improve diagnostic approaches and develop targeted therapies to address the rising burden of AD.

Exosomes are nanosized extracellular vesicles (EVs) secreted by cells, including those in the central nervous system (CNS), into the biofluids. These nanovesicles play vital roles in maintaining the brain microenvironment homeostasis and neural communication, as well as involvement in several pathological pathways related to neurodegenerative diseases [5,6]. Exosomes carry diverse and complex components that serve as unique identifiers of their cells of origin [7]. Among these components, microRNAs (miRNAs) have attracted significant interest in neurodegenerative disease research as they are carried within exosomes and serve as mediators in intercellular communication [8]. These small, single-stranded RNA molecules are selectively sorted and encapsulated within exosomes and transported to target cells, where they regulate gene expression and translation within those cells [9]. The dysregulation of miRNA expression is implicated in various neuropathological mechanisms, including neuroinflammation [10], protein aggregation [11], and impairments in synaptic functions [12], indicating its contribution to mechanisms related to neurodegenerative diseases, including AD. Interestingly, miRNA-containing exosomes released by neural cells can traverse the blood–brain barrier (BBB), allowing the detection of these miRNAs in peripheral body fluids such as blood or cerebrospinal fluid (CSF) [13]. These advantages provide opportunities to understand molecular mechanisms implicated in AD as well as to develop effective diagnostic technologies and targeted therapies.

This review discusses various aspects of AD, including genetic factors, pathophysiological mechanisms, and current diagnostic methods. It also provides information on exosome biogenesis and its constituents, focusing on miRNAs as an exosome component. Additionally, this article provides insights into the important roles of miRNAs encapsulated into exosomes in the pathological mechanisms associated with AD and explores the potential of using exosomal miRNAs as promising tools for diagnosing and treating AD.

## 2. Alzheimer’s Disease (AD)

### 2.1. Genetic Aspects of AD

AD is a complicated and multifactorial neurodegenerative disease. Genetically, AD can be categorized as either sporadic or familial cases. Sporadic AD represents most of the cases (<95%) and typically appears in people aged 65 or older, while >1% are inherited, with a typically earlier onset around age 45 [14]. Familial AD is caused as a result of genetic mutations required in the genes for the metabolism of amyloid-beta (Aβ) peptides, particularly in the amyloid precursor protein (*APP*) gene or presenilin genes (*PSEN1* and *PSEN2*). In contrast, sporadic AD is affected by several risk factors, including genetic, physiological, and environmental components [15,16]. Among the genetic factors, the apolipoprotein E (*APOE*) ε4 allele is the only confirmed genetic risk factor for sporadic AD and is associated with about 50% of those cases [17]. Genetic research has revealed that possessing one of the APOE ε4 alleles triples the risk of AD, while having two of the APOE ε4 alleles raises the risk about twelvefold [18]. Moreover, genome-wide association studies have also identified several common genetic variants that modestly increase AD risk, which are related to immune response and inflammation—such as *CR1* and *TREM2*—suggesting that immune dysfunction and neuroinflammation play roles in AD progression. Lipid metabolism genes, including *CLU* and *ABCA7*, influence Aβ aggregation. Additionally, synaptic function and endocytosis genes—such as *PICALM, CD2AP*, and *BIN1*—affect neurotransmitter release, synaptic plasticity, and Aβ clearance [19].

### 2.2. Pathophysiological Aspects of AD

The pathophysiology of AD is marked by the progressive loss of synaptic connections and subsequent neuronal atrophy in the cerebral cortex [20]. This neurodegenerative process initiates in the hippocampus and entorhinal regions and extends to the frontotemporal cortices of the brain [21]. Postmortem brain examinations of AD patients have revealed key histopathological characteristics that are critical to understanding the disease’s pathophysiology. These characteristics include the abnormal deposition of extracellular amyloid plaques and intracellular neurofibrillary tangles (NFTs), neuro-inflammation, and synaptic dysfunction [22]. Amyloid plaques primarily consist of Aβ deposits produced from the proteolytic processing of APP, a transmembrane protein highly expressed in neural cells. This cleavage is catalyzed by enzymes such as β-secretase, known as beta-site APP cleaving enzyme 1 (BACE1), and presenilin γ-secretase, producing varying lengths of Aβ peptides (typically 40 to 42 amino acids). These Aβ peptides are generally non-toxic [23]. However, aggregate Aβ peptides into oligomer structures are considered highly neurotoxic and represent a critical early event in the AD pathophysiological process [24].

NFTs are another pathological hallmark of AD that appears decades after the initial Aβ emergence. They are primarily derived from the hyperphosphorylation of tau protein [25]. Tau protein, a microtubule-associated protein, is essential for preserving the cytoskeleton, facilitating axonal transport, and supporting neuroplasticity [26]. Under pathological conditions, especially in the presence of excessive toxic Aβ, tau protein becomes hyperphosphorylated. This hyperphosphorylation disrupts tau’s binding to microtubules, forming tau filaments that aggregate into NFTs. These NFTs accumulate in the neuronal cytoplasm, axons, and dendrites, disrupting signal transduction, impairing synaptic transmission, and affecting essential processes such as trophic support and metabolism [27,28]. These disruptions ultimately lead to neuronal death. Studies have shown that Aβ accelerates NFT formation by increasing the activity of protein kinases that phosphorylate tau protein, including cyclin-dependent kinase 5 (CDK5), glycogen synthase kinase 3 (GSK3β), and extracellular signal-regulated kinase 1/2 (ERK1/2) [29].

In addition to the buildup of Aβ plaques and NFT formation, neuroinflammation can contribute to the exacerbation of AD. The buildup of neurotoxic proteins such as Aβ plaques leads to synaptic damage, elevates oxidative stress, and increases the infiltration of microglia cells around the areas where plaques are present [30,31,32]. Microglia, immune cells present in the CNS, are crucial for maintaining the neural environment and protecting from neurotoxic substances. When they encounter protein buildup, microglia become activated and adopt either a pro-inflammatory M1 phenotype or an anti-inflammatory and phagocytic M2 phenotype. These distinct but balanced phenotypes contribute to tissue repair and healing. In AD, microglia are often observed to predominantly display an M1 pro-inflammatory phenotype [33]. This shift reduces amyloid plaque clearance and exacerbates the neuro-inflammatory response, potentially causing irreversible neuronal damage. Although this initial neuro-inflammatory response may be protective, chronic inflammation is considered a crucial factor driving neurodegeneration in AD [34].

Synaptic dysfunction is also a prominent and early characteristic of AD, occurring even before neural degeneration becomes apparent [35]. Early cognitive decline in AD is closely correlated with the reduction in the density of synapses, especially in the hippocampus and medial temporal lobes region of the brain. This reduction in synaptic density reflects the loss and impairment of synapses, disrupting the normal functioning of neural circuits responsible for cognitive processes and leading to the observed decline in cognitive abilities [36,37]. Research on gene expression in AD has revealed alterations in the levels of gene expression associated with synaptic functions, such as those involved in synaptic vesicle trafficking, neurotransmitter release, and the structural components of the synapses [38,39,40]. These alterations in gene expression directly affect synaptic function, an essential aspect of AD and a contributor to cognitive decline.

### 2.3. Clinical Aspects and Diagnosis of AD

The main clinical characteristics of AD include a gradual impairment in functioning and cognitive abilities and are often accompanied by neuropsychiatric symptoms. Nonetheless, patients vary considerably in age of onset, family history, and noncognitive symptoms, such as behavioral or motor abnormalities [41]. Clinicopathological studies have indicated that AD has a long preclinical phase, with amyloid buildup in the brain estimated to begin 10–15 years before cognitive symptoms emerge [3,4]. Once mild cognitive changes are noticeable, the disease progresses from mild impairment to severe dementia at different rates among patients [41].

Memory loss is the predominant symptom of AD, typically following the transitional phase of mild cognitive impairment (MCI), an intermediate state between aging-related cognitive alterations and dementia. MCI is marked by an impairment in one or multiple cognitive abilities without appreciably interfering with daily functioning. MCI is categorized into amnestic and non-amnestic types, with the amnestic form considered as an early stage of AD [42,43]. Although clinical research has indicated that only a small percentage, around 10–15%, of MCI patients progress to AD annually, some patients may maintain stability or even show improvement in their cognitive abilities over time [44].

Diagnosis of AD is a complicated process, and its definitive confirmation can only be confirmed through examination of the postmortem brain. During an individual’s lifetime, AD diagnosis relies on clinical examinations, laboratory tests, and neuropsychological assessments [45,46]. However, these methods have low sensitivity and specificity in early AD detection or providing a definitive diagnosis. Therefore, CSF analysis and neuroimaging of the brain using positron emission tomography (PET) scans are performed to enhance the diagnostic accuracy of AD [45]. Despite their effectiveness, the clinical use of these techniques is limited because they are costly and invasive, making them impractical for screening and frequent monitoring purposes. Consequently, current AD diagnosis primarily depends on cognitive assessments, which are less accurate and often result in delayed or incorrect diagnoses.

Given the current limitations in diagnostic techniques and the critical need for early AD detection, there are significant efforts to develop more reliable methodologies. This involves exploring non-invasive approaches, identifying new biomarkers, and discovering effective treatments for AD. Recently, exosomes have received increasing attention as promising non-invasive tools for identifying novel biomarkers and therapeutic approaches in complex diseases such as AD [47,48,49].

## 3. Exosomes

Exosomes are nanosized vesicles between 50 and 150 nm that carry and transport specific cargo between cells [50]. Neurons and glial cells (astrocytes, oligodendrocytes, and microglia) secrete exosomes that facilitate neuronal communication and regulate several processes, such as immune responses, synaptic transmission, and neuronal development [51]. Moreover, they are implicated in the pathological processes related to neurodegenerative diseases [5,6]. In AD, exosomes participate in the disease’s pathogenesis by spreading pathological agents such as aberrant proteins (e.g., Aβ and tau) and dysregulated miRNAs [52]. These pathological agents can impact the functioning and behavior of recipient cells, potentially exacerbating the pathological processes associated with AD.

### 3.1. Biogenesis and Uptake of Exosomes

**Figure 1:**
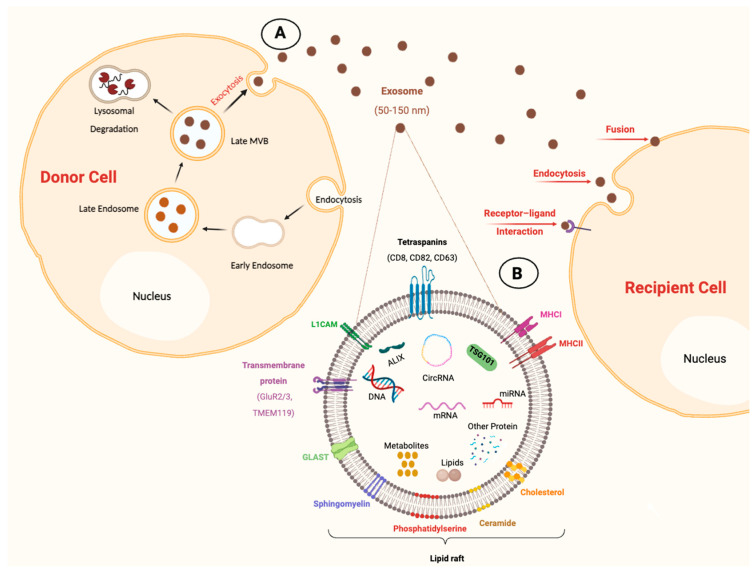
Illustration of exosome biogenesis and its constituents. (**A**) Exosomes are generated during the formation of early endocytic vesicles (early endosomes), followed by inward invagination of this endosomal membrane and the formation of multivesicular bodies (MVBs) (late endosomes). Some MVBs progress to late stages and subsequently can either be fused with lysosomes, causing cargo degradation, or be fused with the plasma membrane of cells, secreting their content as exosomes into extracellular space. Exosomes can then interact with recipient cells by membrane fusion, endocytosis, or receptor–ligand interactions. (**B**) Exosomes are enveloped in a phospholipid bilayer with various surface components. These components include tetraspanins (CD8, CD63 and CD82); cell adhesion molecules such as L1 cell adhesion molecule (L1CAM); transmembrane proteins such as 119 (TMEM119) and glutamate receptor (GluR2/3); major histocompatibility complex molecules (MHCI and MHCII); transporter proteins such as glutamine aspartate transporter (GLAST); and lipid rafts. The internal contents of exosomes consist of various biological species, including RNA molecules (circRNA, mRNA, and microRNA), proteins, DNA, lipids, and metabolites. Additionally, exosomes can contain specific markers, such as tumor susceptibility gene 101 (TSG101), heat shock proteins (HSPs), and apoptosis-linked gene 2-interacting protein X (ALIX), which are commonly utilized as markers for exosomes (image created with BioRender.com).

Exosome biogenesis is a complicated process involving multiple sequential steps, as illustrated in Figure 1. It begins with forming an early endosome by endocytosis of the cell membrane component, then progresses into a late endosome that finally matures into multivesicular bodies (MVBs). MVBs are characterized by containing multiple intraluminal vesicles (ILVs) resulting from the inward invagination of the late endosome into its lumen. During the formation of ILs, the proteins and lipids of the cell membrane are integrated into the invaginating membrane, while cellular constituents are sorted and packaged into these vesicles [53]. The process of cargo sorting within the ILVs is highly regulated and involves several pathways. One of the primary pathways is the endosomal sorting complex required for transport (ESCRT) machinery, which involves ESCRT proteins and associated factors, including lipids (e.g., ceramide) and proteins such as tetraspanins (CD9, CD63, CD81), vacuolar protein sorting-associated protein 4 (VPS4), and apoptosis-linked gene 2-interacting protein X (ALIX). Besides the ESCRT-dependent pathway, cells can also use ESCRT-independent mechanisms for sorting cargo into ILVs. These mechanisms involve lipids such as ceramide and tetraspanins, which help sort specific proteins and lipids into ILVs [54]. Once ILVs are formed within MVBs, these vesicles can follow different pathways. Some MVBs fuse with lysosomes, resulting in their cargo degradation as part of the endolysosomal recycling pathway [55]. Alternatively, MVBs can move to the plasma membrane and undergo exocytosis, releasing their ILVs into extracellular space as exosomes [56].

Exosomes can interact with recipient cells after being released from parent cells through three primary mechanisms: endocytosis, membrane fusion, or receptor–ligand interactions, as shown in Figure 1. During endocytosis, recipient cells take up exosomes either by non-specific or receptor-mediated processes. Once internalized, they release their content into the intracellular environment of the recipient cells. Exosomes can also directly fuse with cell membranes to release their cargo to recipient cells. Moreover, exosomes can attach to the recipient cell’s membrane without internalization, thus influencing downstream signaling pathways [57,58].

### 3.2. Constituents of Exosomes

EVs, including exosomes, carry a cargo that consists of a diverse array of bioactive molecules derived from their parent cells, including proteins, various RNA species (such as mRNA, miRNA, and lncRNA), DNAs (including mtDNA, ssDNA, and dsDNA), lipids, and metabolites (Figure 1). This cargo varies according to the EVs’ cellular origins, the type of parent cells, and the surrounding physiological conditions. For instance, exosomes originating from endosomal compartments exhibit enrichment in proteins associated with MVB biogenesis, such as TSG101 and Alix, as well as tetraspanins [59]. Several studies used these protein markers to distinguish exosomes from other EVs besides simple size-based ones [60].

In addition to the typical protein cargo, exosomes express specific protein markers associated with their parent cells and functions. These markers can be utilized to identify and characterize different subtypes of exosomes. For instance, exosomes derived from neurons contain synaptic proteins, such as glycosylphosphatidylinositol (GPI)-anchored prion protein, L1 cell adhesion molecule (L1CAM), and glutamate receptor subunits (e.g., GluR2/3) [61]. Exosomes secreted by astrocytes highly express the astroglia surface protein glutamine aspartate transporter (GLAST), which maintains glutamate clearance and neurotransmitter homeostasis [62]. Microglia secrete exosomes that express specific proteins such as transmembrane protein 119 (TMEM119) and CD11b [63]. These protein markers are considered identifiers of the origin of neural cells and were used in several studies to isolate CNS-specific exosomes using antibodies targeting these proteins [61,62,63].

Exosomal cargo also varies based on the status of the cell and its surrounding microenvironment. Changes in the local microenvironment, such as protein aggregations, inflammation, or cell activation, can directly alter the cellular processes within the cell and, subsequently, the cargo loaded into exosomes. This alteration in exosomal cargo can reflect the molecular mechanisms and biological processes in the cell. Gao et al. found that microglia activated by glutaminase C protein release exosomes with distinct miRNA compositions compared to exosomes released from resting microglia [64]. Additionally, neuron-derived exosomes isolated from individuals with AD have exhibited alterations in protein compositions compared to those from healthy individuals [65]. These changes in exosomal cargo can provide information about intracellular and extracellular changes related to the physiological and pathological processes.

## 4. miRNAs

**Figure 2:**
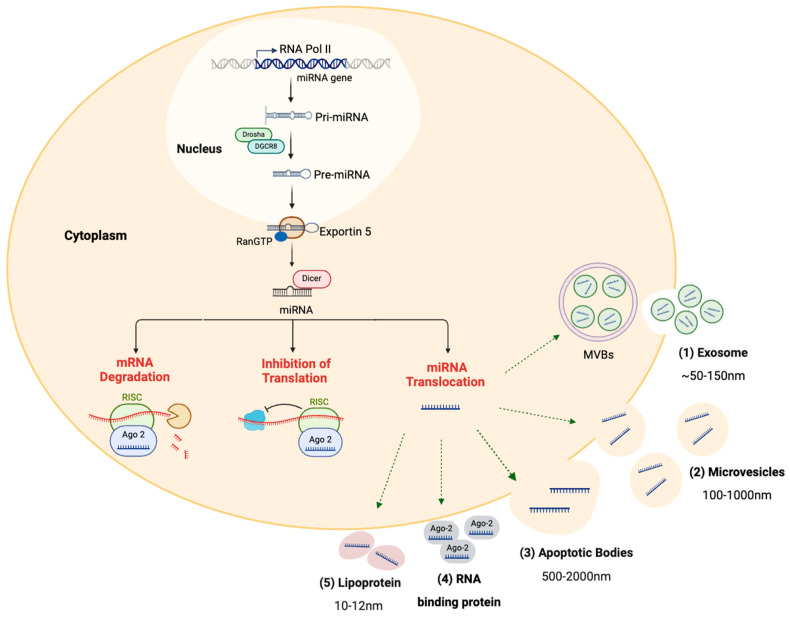
Illustration of miRNA biogenesis and their biological functions. The miRNA biogenesis process initiates in the nucleus of the cell, where miRNA gene transcription is carried out by RNA polymerase II, producing primary miRNAs (pri-miRNAs) that are relatively long, capped, and polyadenylated transcripts. The pri-miRNAs undergo cleavage by the Drosha–DGCR8 complex to form hairpin-shaped structures called precursor miRNAs (pre-miRNAs), which are then transported to the cytoplasm through the exportin-5/Ran-GTP complex. After that, the Dicer enzyme further processes pre-miRNAs into double-stranded miRNAs, which then unwind to form mature miRNAs. Mature miRNAs have two fates. First, they can be loaded with Ago2 protein into the RNA-induced silencing complex (RISC) and bind to specific sequencing in the target mRNA, leading to either their degradation or suppression of translation. Second, they can be secreted into extracellular circulation through (1) packaging into exosomes, (2) enclosing within MVs, (3) accumulating within apoptotic bodies, (4) binding to Ago2 protein, or (5) binding to lipoproteins (image created with BioRender.com).

miRNAs are short, non-coding RNA molecules, typically 18–25 nucleotides long, and are crucial in regulating gene expression. Biogenesis of miRNAs begins with transcripts of miRNA genes in the cell nucleus. Then, these miRNA transcripts undergo several processing steps to produce mature miRNA molecules (Figure 2). Mature miRNAs primarily function as post-transcriptional gene-silencing agents by binding to specific target sites at the 3′ untranslated regions (3′ UTRs), 5′ UTR, or coding regions of target messenger RNAs (mRNAs). The degree of complementarity between the miRNA-specific seed sequences (two–eight nucleotides at the 5′ end) and target sequences on the mRNA determines the specific regulation type that occurs. Perfect complementarity results in mRNA degradation, while imperfect complementarity inhibits translation [9]. Functionally, a single miRNA can regulate multiple cellular pathways by interacting with various mRNA targets. Conversely, multiple miRNAs can collaborate to regulate a specific pathway by targeting particular mRNA molecules [66].

Moreover, research has demonstrated that miRNAs can also upregulate gene expression through a mechanism called RNA activation. In this mechanism, miRNAs interact with gene promoters or enhancers to activate transcription [67,68]. This process occurs after the miRNAs have been transported from the cytoplasm to the nucleus.

According to information from the miRBase (v22) and GENCODE databases, there are approximately 3000 mature miRNAs and over 200,000 transcripts, which include variations in isoforms. Studies have suggested that miRNAs modulate over 60% of protein-coding genes within the human genome [69]. Furthermore, around 70% of known miRNAs are expressed in brain regions in a temporally and spatially regulated manner [70]. miRNAs play diverse roles in the brain’s development and function, coordinating complex molecular processes to support proper neural development, synaptic connectivity, and the maintenance of neural stem cell populations [71]. Neuronal and glial cells have distinct miRNA profiles essential for their development, homeostasis, and functions. The dysregulation of miRNA expressions and subsequent alterations in gene expression are implicated in different pathological mechanisms in neurodegenerative diseases, including those related to the pathogenicity of AD [72,73].

Besides their intracellular role in regulating gene expression, miRNAs also function as mediator signals between cells. Cells can release miRNAs into extracellular fluids in different ways, including packaging them into exosomes, enclosing them within microvesicles (MVs), accumulating them within apoptotic bodies, or binding them to proteins [e.g., Argonaute 2 (Ago2) or lipoproteins]. These circulating miRNAs in the extracellular space are called circulating miRNAs (Figure 2) [9]. Target cells internalize those circulating miRNAs and use them to modulate several cellular functions, coordinate with other cells, or biological homeostasis [74].

### 4.1. Characteristics of Exosomal miRNAs

miRNAs in exosomes have gained considerable research attention, particularly in studies of neurodegenerative diseases. The encapsulation of miRNAs within exosomes provides a protective environment that enhances their stability, protecting them against degradation by extracellular enzymes or unfavorable environmental factors. Moreover, the lipid bilayer membrane that envelops exosomes and their nanosize enables them to cross the BBB and be transported to distant cells through circulation in various biological fluids. The existence of exosomal miRNAs in biofluids presents a non-invasive approach to readily access and analyze miRNA profiles, which could yield valuable insights for AD diagnosis and monitoring [75].

Research has indicated that the selection of exosomal miRNAs and their packaging into exosomes are not random processes but rather exhibit specificity, which depends on the type of cell and/or the surrounding microenvironment conditions. Although the exact mechanisms of sorting miRNAs within exosomes are unclear, studies have proposed that exosomes use two key mechanisms: selective and nonselective sorting. Nonselective sorting occurs when miRNAs are packaged into exosomes without specific recognition, potentially due to their abundance in the cytosol. Conversely, selective sorting involves specific proteins recognizing and binding to particular sequence motifs in miRNAs. These sorting mechanisms can vary based on the cellular composition and the origins of exosomes, which makes them an interesting strategy for diagnosing AD early and accurately based on cell-type signatures [76].

### 4.2. Exosomal miRNAs: Sources, Extraction, Profile Analysis, and Target Identification in AD

#### 4.2.1. Sources of Exosomal miRNAs

**Figure 3:**
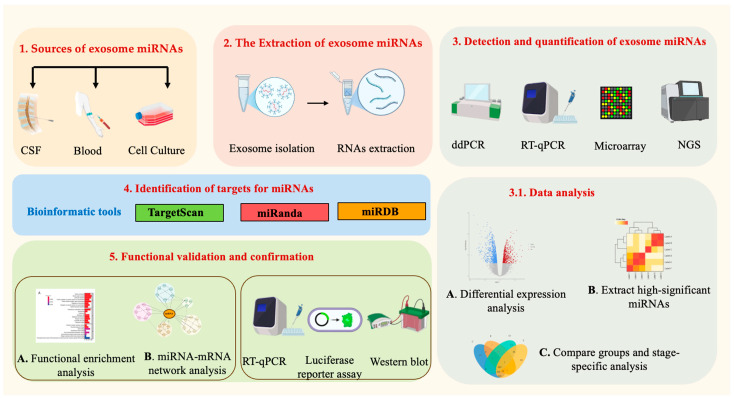
Workflow for analyzing exosomal miRNAs in AD diagnosis (image created with BioRender.com).

Exosomes are released in most bodily fluids including blood, CSF, urine, and various tissue fluids [77]. Researchers are studying the contents of exosomes, including miRNAs, in different biological fluids and in vitro culture media to identify potential therapeutic targets and develop biomarkers for neurodegenerative diseases, including AD (Figure 3). Recent studies have concentrated on establishing relevant biomarkers for AD by investigating the packaging of exosomal miRNA, focusing on their roles in CSF and blood [78,79].

The direct interaction of CSF with the CNS enables it to reveal biochemical changes and ongoing neurodegenerative processes in the brain, thereby offering valuable insights into AD mechanisms [80]. Although studying exosomal miRNAs in CSF is important for understanding the pathogenicity of AD, their potential as biomarkers and therapeutic targets has some limitations. Firstly, collecting CSF samples is challenging because it requires a lumbar puncture, a procedure that can be uncomfortable and carries risks to patients including infection or headaches. These concerns may limit the number of participants in AD studies, potentially impacting the generalizability of the findings. Secondly, the volume of CSF that can be collected is limited, which complicates comprehensive analyses and makes repeated sampling and routine screening for AD more challenging. Furthermore, the sensitivity for detecting low-abundance miRNAs in exosomes isolated from CSF is constrained by the challenges in obtaining sufficient quantities of exosomal miRNA. Thirdly, contamination with blood or other substances may occur during CSF collection, potentially interfering with the findings. Consequently, these factors can compromise the reliability of using exosomal miRNAs as biomarkers for AD.

In addition to CSF, blood-based biomarkers are increasingly utilized for diagnosing and predicting a range of neurodegenerative diseases. CNS cell-derived exosomes can diffuse bidirectionally through the BBB between the bloodstream and brain parenchyma, making them promising tools for diagnosing and treating AD. Blood sampling is non-invasive and easily accessible, and the availability of larger sample volumes and higher enrichment of exosomal miRNAs enhances biomarker screening, improving both sensitivity and specificity for disease detection [81]. However, several limitations exist to utilizing exosomal miRNA derived from blood for clinical applications in AD. A major challenge is the heterogeneity in exosomes, as blood comprises various populations that originate from different organs and tissues. This diversity can hide primary AD-specific changes in exosomal miRNAs by introducing non-specific secondary changes, making it more difficult to identify relevant biomarkers. Nonetheless, advancements in technology have made it possible to address this challenge. As mentioned earlier, neural-derived exosomes can be isolated using antibodies that target exosomal proteins specific to neuronal or neuroglial cells. Furthermore, focusing on studying AD-specific biomolecules such as Aβ, hyperphosphorylated tau protein, and specific miRNAs (e.g., miR-193b and miR-125b-5p), which are associated with AD, can reduce the impact of heterogeneity and increase analysis specificity [82]. Additionally, advanced machine learning algorithms can detect patterns and relationships among various biomarkers, thereby improving analytical accuracy and deepening our understanding of the mechanisms underlying AD. Consequently, integrating multi-omics analysis with large-scale studies involving a substantial number of participants can yield a more comprehensive understanding of AD and potentially identify novel biomarkers that enhance early diagnosis and treatment strategies for the disease.

In contrast to research focused on exosomal miRNAs in biofluids, in vitro models and animal models of AD offer valuable platforms for studying and manipulating exosomal miRNAs under specific conditions. By altering the expression of specific miRNAs in donor cells, we can modify the expression of miRNAs into exosomes of recipient cells and explore their functions and mechanisms related to AD. Despite in vitro disease models being helpful for many functional analyses, they cannot fully replicate the intricate microenvironment of living organisms. Therefore, it is essential to validate findings obtained in vitro by confirming them through studies using animal models or human organoid models of AD.

#### 4.2.2. The Extraction of Exosomal miRNAs

The presence of various miRNAs that bind to proteins in bodily fluids necessitates the initial isolation of exosomes before extracting and purifying miRNAs derived from these exosomes. The two predominant approaches currently utilized for the isolation of exosomes are ultracentrifugation (UC) and commercially available precipitation-based kits [81]. UC is widely used and relies on differential centrifugation to separate exosomes based on size and density. However, this method has limitations, including time consumption and difficulty in excluding non-exosomal contaminants [83]. On the other hand, precipitation kits offer a faster alternative and can handle larger sample volumes, though they may suffer from purity issues due to the co-isolation of other EVs [84]. Both methods yield exosomes of similar purity, but UC typically produces fewer exosomes. Other methods, such as size exclusion chromatography and immunoaffinity isolation, are less common due to challenges such as low yield and high costs. Recently, immunoprecipitation techniques that target specific cell types have emerged, enhancing the specificity of CNS-derived exosome isolation and facilitating studies on their roles in AD [84].

After isolating exosomes, extracting miRNA is essential for enhancing purity and minimizing losses during separation from EVs. Two primary methods for miRNA isolation are TRIzol and commercial kits such as ExoQuick. TRIzol maintains RNA integrity during lysis but may leave behind phenols and salts, affecting downstream analyses. To improve efficiency, column purification with TRIzol and solid-phase extraction methods using magnetic beads are effective. Commercial kits often yield higher miRNA quantities from biological fluids compared to UC methods, along with providing more consistent miRNA profiles [84,85]. This enhanced performance is likely a result of the better recovery efficiencies of exosomal miRNAs offered by these kits [86].

The consistency of miRNA profiles is a critical consideration in AD research, as it influences choosing effective isolation methods and the interpretation of data. Studies have shown a strong correlation in miRNA expression between exosomes isolated using UC and those obtained from commercial kits [84]. However, statistical differences in 17 specific miRNAs indicate discrepancies in the components of exosomes isolated by these methods. Furthermore, while a general correlation in exosomal miRNA profiles exists, the recovery rates of specific miRNAs (let-7d, miR-16, and miR-25) are significantly different between commercial isolation kits [83]. These variations can largely be attributed to the technical differences among the kits. Consequently, the lack of standardized procedures for exosome and exosomal miRNA extraction complicates efforts to investigate miRNA profiles in AD, identify potential biomarkers, and understand the pathological and therapeutic roles of exosomal miRNAs.

#### 4.2.3. Profile Analysis of Exosomal miRNAs

The detection and quantification of exosomal miRNAs are crucial for advancing AD research, as these molecules can serve as potential biomarkers for early diagnosis, progression monitoring, and therapeutic targets. Several methods are employed for the detection and quantification of EV-miRNAs. One commonly used approach is reverse transcription–quantitative polymerase chain reaction (RT-qPCR), which allows for the specific amplification and quantification of miRNA targets. RT-qPCR can be combined with pre-amplification steps to enhance the sensitivity of detection. Additionally, next-generation sequencing (NGS) techniques provide comprehensive profiling of exosomal miRNAs, allowing for the identification of novel miRNAs and assessment of their expression levels. NGS-based methods can also provide information on the miRNA sequence, facilitating the discovery of functional miRNA targets. Other emerging technologies, such as digital droplet PCR (ddPCR) and microarray-based approaches, offer alternative strategies for quantifying and profiling exosomal miRNAs. These methods provide high sensitivity, specificity, and multiplexing capabilities, enabling the detection and quantification of miRNAs in complex biological samples. As the field advances, developing standardized protocols, data analysis pipelines, and validation of exosomal miRNA biomarkers will be crucial for their clinical translation and integration into diagnostics and therapeutic strategies for AD.

#### 4.2.4. Identification of Targets for Exosomal miRNAs

In general, miRNAs function by binding to target mRNA molecules through partial base pairing, which leads to the repression of translation and/or degradation of mRNA. Although the experimental strategies used to identify miRNA targets may vary, the underlying logic behind these strategies is generally consistent and standardized [13]. Two basic steps are involved in identifying miRNA targets: screening for putative targets of miRNA and validating candidate transcripts associated with miRNA. Putative miRNA targets for AD biomarkers can be identified using a combination of experimental and computational approaches. Experimental approaches use high-throughput sequencing techniques to profile miRNA expression levels in different biological samples from AD patients and healthy controls, such as CSF, plasma, or brain tissue. This can identify differentially expressed miRNAs that may be involved in AD pathology. Once candidate miRNAs are identified, their potential targets can be predicted using computational algorithms such as TargetScan, miRanda, and miRDB, which can identify potential mRNA targets based on miRNA-mRNA complementarity.

Candidate transcript validation of miRNA-target interactions is also crucial in AD biomarker research. This can be performed using techniques including qRT-PCR, luciferase reporter assays, or Western blot analysis, which can confirm the interaction between the miRNA and its predicted target mRNA and provide insight into the mechanisms of miRNA-mediated regulation in AD pathology. In addition to these techniques, network analysis and pathway analysis can also be used to identify putative miRNA targets by examining the functional relationships between differentially expressed miRNAs and their predicted target genes in the context of AD pathology.

### 4.3. Roles of Exosomal miRNAs in the Pathogenicity of AD

**Figure 4:**
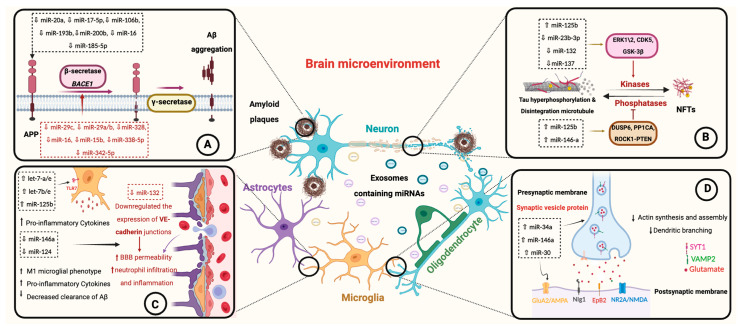
Involvement of miRNAs in various mechanisms of AD pathology, including (**A**) amyloid-beta protein (Aβ) depositions, (**B**) neurofibrillary tangle (NFT) formations, (**C**) neuroinflammation, and (**D**) synaptic dysfunction (image created with BioRender.com).

Exosomes have attracted significant interest in AD research due to their role in neural communication and their ability to transport miRNAs between cells. Exosome-associated miRNAs have been implicated in various AD pathological mechanisms, such as the production of Aβ, tau pathology, neuroinflammation, and synaptic dysfunction (Figure 4 and Table 1). The transmission of miRNAs via exosomes from affected cells to neighboring or distant cells can target and regulate multiple genes associated with pathological processes, thereby influencing the pathogenesis of AD.

#### 4.3.1. miRNAs in Aβ Production

Dysregulation of APP processing is associated with AD development, as disturbances in this process can generate Aβ peptides. These peptides accumulate and subsequently form amyloid plaques that deposit between neural cells of AD brains. Research has demonstrated that miRNAs modulate *APP* mRNA expression by directly binding to the 3′ UTR, thereby modulating its stability and translation. Members of the miR-20a family (miR-17-5p, miR-20a, and miR-106b) have been demonstrated to downregulate APP mRNA expression and inhibit abnormal Aβ accumulation in neuronal cell lines [87]. Among these family members, miR-20a-5p has been observed to be significantly reduced in the exosomes isolated from the blood of individuals with sporadic AD. This reduction in exosomal miR-20a-5p levels could contribute to Aβ plaque formation and the cognitive impairment observed in AD [88]. miR-193b is another miRNA that suppresses *APP* mRNA expression and promotes the degradation of its mRNA. In AD patients, an inverse correlation has been observed between the levels of the amyloidogenic Aβ42 peptide and miR-193b encapsulated into exosomes within the CSF, indicating that exosomal miR-193b may serve as a biomarker to detect the pathological changes related to AD [89,90]. Similarly, miR-16 is another miRNA identified as a suppressor of *APP* mRNA expression, as confirmed in various types of brain cells [91]. Interestingly, miR-16 levels in exosomes isolated from CSF were lower in patients with early-onset AD (YOAD) than in those with late-onset AD (LOAD) [92]. This suggests the direct involvement of miR-16-5p in regulating APP transcription, which is particularly relevant to early-onset AD pathogenesis. Furthermore, miR-185-5p acts as an inhibitor of APP transcript, and its expression has been dramatically reduced in serum-derived exosomes of AD patients [93,94].

The BACE1 enzyme is considered the major cleavage enzyme of APP, marking the initial step in Aβ formation, and its levels and activity were elevated approximately two-fold in the brains of patients with AD [95]. Several miRNAs have been demonstrated to regulate *BACE1* mRNA expression in various neural cell lines by binding to its 3′UTR, thereby influencing BACE1 protein levels. For instance, the miR-29 family is a highly studied miRNA group known for regulating *BACE1* mRNA expression and affecting its translation. This family consist of three miRNAs: miR-29a, miR-29b1, and miR-29c [96,97]. In AD brains, a major downregulation in the miR-29a/b-1 cluster has been observed, and its downregulation is associated with increased BACE1 protein levels [98,99]. Interestingly, the downregulation of miR-29c was also found in CSF-derived exosomes of individuals with AD [92]. miR-342-5p is another miRNA that regulates *BACE1* mRNA expression, and its downregulation was associated with cognitive decline severity in AD patients [100]. The downregulation of miR-342-5p was also observed in blood-derived exosomes of AD patients. Further experiments confirmed that exosomal miR-342-5p can protect recipient neurons against neuronal toxicity induced by Aβ deposition by inhibiting the expression of *BACE1* mRNA [101]. Similarly, miR-15b acts as a suppressor of *BACE1* mRNA expression, and its upregulation may reduce Aβ aggregation and enhance the survival of Aβ-treated SH-SY5Y cells [102,103]. miR-328, miR-338-5p, and miR-16 also act as negative regulators of *BACE1* mRNA and have been found to be downregulated in exosomes derived from blood and CSF of individuals with AD [78,92,104,105,106,107].

#### 4.3.2. miRNAs in Tau Pathology

Increased tau phosphorylation and the subsequent formation of NFTs inside neural cells are key factors causing cognitive decline in AD patients. The aberrant in tau phosphorylation results from an imbalance in regulating tau phosphorylation and dephosphorylation, which involves the activation of kinases and phosphatases associated with tau protein. miRNAs play significant roles in regulating tau phosphorylation and contribute to tauopathy in AD. One such miRNA is miR-125b, which exhibits high expression in neural cells and cells and significantly regulates tau protein phosphorylation [108]. Several studies have observed overexpression of miR-125b in the brain and biofluids of AD patients, and this elevation in miR-125b was correlated with tau toxicity-induced cognitive impairment [92,108,109,110]. Overexpression of miR-125b in neurons led to increased tau phosphorylation by activating kinases (ERK1/2 and CDK5/P35) and simultaneously suppressed the activity of phosphatases [dual-specific phosphatase 6 (DUSP6) and protein phosphatase 1 catalytic subunit alpha (PP1CA)]. This imbalance between kinases and phosphatases caused tau hyperphosphorylation and apoptosis of neurons [107,108]. Moreover, miR-125b can enhance tau pathology by activating GSK-3β, an essential kinase contributing to tau phosphorylation [109]. Elevated GSK-3β activity was observed in the brains of AD patients, and its elevation has been implicated in tau pathology development and correlated with cognitive decline [111,112]. Experimental evidence from mouse models of dementia has confirmed that the upregulation of miR-125b increases GSK-3β activity, suggesting involvement in the formation of NFTs and progression of tau-mediated pathology [109]. miR-137 is abundantly expressed in the cerebral cortex and plays a significant role in synaptic functions and neuronal differentiation. miR-137-3p is another miRNA that targets GSK-3β activity and modulates its expression. Studies in AD cells and animal models have shown that downregulation of miR-137-3p leads to an imbalance in tau phosphorylation through increased GSK-3β mRNA expression [113]. In contrast, miR-23b-3p suppresses *GSK-3β* mRNA expression and effectively reduces tau phosphorylation in cell cultures. However, the expression of miR-23b-3p was found to be decreased in the blood of AD individuals, and this reduction is negatively associated with the degree of tau phosphorylation as the disease progresses [112]. Additionally, the levels of miR-23b-3p encapsulated within exosomes have been lower in the blood of AD patients than in healthy individuals [107]. The miR-132/212 cluster is a highly studied and well-characterized group of miRNAs playing a significant role in aberrant tau phosphorylation in various neurodegenerative diseases [114]. miR-132 is one of the well-confirmed miRNAs that are downregulated in the brains of AD patients. Importantly, this reduction in miR-132 levels has been observed even before the onset of neuronal loss and is strongly correlated with the severity of tau pathology related to AD [115,116]. Multiple studies have shown that miR-132 protects nerve cells against forming NFTs and synaptic dysfunction through various mechanisms. These mechanisms involve miR-132 targeting *tau* mRNA, inhibiting GSK3β activity, or enhancing tau protein cleavage and degradation [117,118]. An experimental study demonstrated that reducing miR-132 levels increases total and phosphorylated tau protein levels in a transgenic mouse model of AD. Conversely, restoring normal miR-132 levels in these mice improves tau pathology and cognitive functions [117]. Moreover, miR-132 indirectly enhances NFT formation by targeting the expression of inositol 1,4,5-trisphosphate 3-kinase B (ITPKB), a kinase that activates the ERK1/2 pathway and subsequently promotes tau phosphorylation [119,120]. Also, miR-146a indirectly promotes tau phosphorylation in neuronal cells by suppressing the expression of rho-associated coiled-coil-containing protein kinase 1 (ROCK1), which in turn activates GSK3β indirectly through targeting the phosphatase and tensin homolog (PTEN). Inhibition of miR-146a decreased phosphorylated tau levels and improved memory dysfunction in mice models of AD [121].

#### 4.3.3. miRNAs and Neuroinflammation

Neuroinflammation is one factor contributing to the development of AD and greatly influences its progression. The chronic activation of inflammatory immune cells results in the excessive production of inflammatory factors, further advancing the disease. Although innate immune cells are primarily responsible for mediating neuroinflammation, recent studies suggest that blood cells can penetrate the BBB and participate in the inflammatory responses in AD [122].

miRNAs are essential in regulating neuroinflammation in the CNS by targeting genes involved in immune responses. The Let-7 family, an evolutionarily conserved group of miRNAs, significantly modulates various inflammatory processes, including microglia activation and polarization, astrocyte differentiation, and pro-inflammatory cytokine expression [123,124]. Let-7 miRNAs can function as damage-associated molecular patterns (DAMPs) and ligands for toll-like receptor 7 (TLR7) on neural cells, including microglia, leading to its activation and enhancement of neuroinflammation [125,126]. Additionally, they can regulate inflammation by directly targeting cytokine production, including interleukin (IL)-6 and IL-10 [127]. Neurons release proteins belonging to the Let-7 family, which have been detected to be overexpressed in neuronal exosomes in AD patients [128]. Conversely, miR-125b exacerbates neuroinflammation by enhancing the production of pro-inflammatory cytokines, including tumor necrosis factor-alpha (TNF-α), IL-1β, and IL-6 [129]. Inhibiting miR-125b decreases the synthesis and production of pro-inflammatory cytokines, thus alleviating the inflammatory response associated with AD [130]. miR-146a is another essential regulator of neuroinflammation, and it is highly expressed in neural cells [131]. Upregulation of miR-146a beneficially reverses microglia polarization towards the M2 phenotype, which, in turn, enhances the clearance of Aβ plaques and reduces pro-inflammatory cytokine production. Administering miR-146a reduced Aβ accumulation, mitigated neuroinflammation, reduced neuronal death, and improved cognitive functions in APP/PS1 transgenic mice [132]. Furthermore, miR-146a is a protective agent in alleviating astrocytic inflammation by inhibiting NF-κB activation, a well-known pro-inflammatory signaling pathway [133]. However, despite its positive effects, miR-146a was found to be downregulated in exosomes derived from the blood of individuals with AD [134].

Evidence suggests that BBB disruption is a common feature of neuroinflammation-mediated neurodegeneration [122]. The BBB is a highly selective barrier composed of endothelial cells, tight junctions, and supporting cells, which collectively work to maintain homeostasis within the CNS. In AD, changes in the integrity of tight junctions between endothelial cells are prevalent pathological characteristics observed in over 90% of AD brains during autopsy [135]. These changes in tight junction integrity may enhance immune cell infiltration and contribute to the amplified neuro-inflammatory processes associated with AD. miRNAs regulate the expression of molecules essential for maintaining endothelial junctions, consequently affecting BBB function [136]. For instance, miR-132 regulates vascular endothelial (VE)-cadherin expression, a key molecule in the formation and stability of adherent junctions between endothelial cells [137]. Interestingly, neurons release exosomes containing miR-132, which are internalized by brain endothelial cells, enhancing the expression of functional and mature miR-132. This process leads to increased levels of VE-cadherin and improved integrity of the BBB [138]. Conversely, in AD, evidence indicates reduced miR-132 levels in neuronal exosomes [139], which could decrease VE-cadherin expression and contribute to BBB dysfunction [140].

#### 4.3.4. miRNAs and Synaptic Dysfunction

Synapses are the basic brain communication units that allow neural cells to transmit and receive signals. The structural and functional integrity of these synapses is critical for normal neurotransmission and the proper functioning of synaptic and cognitive processes. In AD, disturbances in synaptic function and compromised synaptic plasticity, particularly in the earlier stages, lead to cognitive deterioration. Research indicates that miRNAs within synaptic structures may be implicated in disrupting neurotransmission and plasticity processes, consequently contributing to cognitive deterioration in AD patients [141].

miRNAs directly impact synaptic function by modulating the gene expression of essential proteins within the synaptic structure, including ion channel subunits, surface receptors, and synaptic vesicle components critical for neurotransmission. miR-34 is one of the miRNAs essential in synaptic functions, contributing to synapse formation and connections between nerve cells [142]. miR-34a affects synaptic function by downregulating the expression of the NR2A subunit of the N-methyl-D-aspartate (NMDA) receptor at the postsynaptic compartment of neural cells. The NMDA receptor is a glutamatergic receptor essential in synaptic plasticity and memory formation, especially in the hippocampus [143]. miR-34a also negatively affects proteins involved in synaptic transmissions by downregulating the expression of vesicle-associated membrane protein 2 (*VAMP2*) and synaptotagmin 1 (*SYT1*). VAMP2 and SYT1 proteins are essential in synaptic vesicle trafficking and releasing of neurotransmitters at the presynaptic compartment of neural cells [39]. Similarly, miR-146a-5p reduces the levels of synaptic proteins by targeting *SYT1* and neuroligin 1 (*Nlg1*) [38]. Nlg1 is a postsynaptic protein contributing to dendritic spine formation and the stability of synapses [144]. Microglia release exosomes containing miR-146a-5p, which then bind to the mRNA of *SYT1* and *Nlg1* in neurons, thereby inhibiting their expression. The downregulation of *SYT1* in the presynaptic compartment disrupts neurotransmitter release and impacts synaptic transmission. On the other hand, the downregulation of *Nlg1* in the postsynaptic compartment interferes with the establishment and stability of excitatory synapses [38]. miR-30b is another miRNA impairing synaptic and cognitive functions through different mechanisms. It directly binds to and targets the mRNA of glutamate receptor subunit 2 (GluA2), an AMPA receptor subunit crucial in synaptic strength regulation and normal synaptic transmission [145]. Additionally, miR-30 downregulates the expression of ephrin type-B receptor 2 (*ephB2*) and sirtuin 1 (*SIRT1*), which protect glutamate receptors and maintain synaptic function. However, miR-30b has been upregulated in the blood of AD patients and mice models of AD. This elevation of miR-30b has been associated with impaired basal synaptic transmission and decreased dendritic spine density in the hippocampus of transgenic mice, which may cause a decline in learning and memory abilities related to AD [145].

**Table 1:** Some exosomal miRNAs that play roles in the pathogenesis of AD.

The Ultimate Target	MiRNA	Functions	Animal Model/Cell Line	Patient Samples		Ref.
				Brian	Peripheral Body Fluid	
Aβ production	miR-20a	Downregulated Aβ accumulation by binding to *APP* mRNA, thereby decreasing *APP* mRNA expression.	HeLa, Neuro2A, and SK-N-SH cells	Anterior temporal cortex or Cerebellum	Blood-derived exosomes	[87,88]
	miR-193b		APP/PS1 mice		CFS- and blood-derived exosomes	[89,90]
	miR-16		Rat neuronal PC12 and primary hippocampal neuron cells		CFS-derived exosomes	[91,92]
	miR-185-5p		APP/PS1 mice/N2a (Aβ) cells		Blood-derived exosomes	[93]
	miR-29c	Downregulated Aβ accumulation by binding to *BACE1* mRNA, thereby decreasing *BACE1* mRNA expression.	SH-SY5Y (Aβ) cells	Frontal cortices	CFS-derived exosomes	[80,96]
	miR-29a/b		SK-N-SH cells	Cortex		[98]
	miR-328		APP/PS1 mice/N2a (Aβ) cells		CFS-derived exosomes	[78,104]
	miR-16		SH-SY5Y (Aβ) cells		CFS-derived exosomes	[92,146]
	miR-15b		SH-SY5Y (Aβ) and HEK293T cells	Frontal cortices	Blood-derived exosomes	[102,147,148]
	miR-338-5p		5XF-AD TG mice/primary hippocampal neurons cells	Hippocampus	Blood-derived exosomes	[105,107]
	miR-342-5p		HT22 cells		Blood-derived exosomes	[101]
Tau Pathology	miR-125b	Upregulated Erk1/2 and CDK5/P35 kinases and downregulated DUSP6 and PPP1CA phosphatases, enhancing tau hyperphosphorylation.	Primary hippocampal and cortical neuron cells	Frontal cortex	CFS- and blood-derived exosomes	[92,108,110]
		Enhanced tau hyperphosphorylation by activating GSK-3β.	APP/PS1 mice/N2a cells			[109]
	miR-23b-3p	Reduced phosphorylation of tau protein by suppression of GSK-3β activity.	APP/PS1 and SAMP8 mice; APPswe cells		Blood-derived exosomes	[107,149]
	miR-137-3P	Enhanced tau hyperphosphorylation by activating GSK-3β.	APP/PS1 mice/SH-SY5Y (Aβ) cells	Hippocampus		[113]
	miR-132	Reduced tauopathies by targeting *Tau* mRNA, inhibiting GSK3β, or enhancing tau protein cleavage and degradation.	3xTg-AD mice/primary hippocampal and cortical neuron cells	Temporal cortex, prefrontal cortex and hippocampus	Blood-derived exosomes	[117,118,139]
	miR-146-a	Enhanced tau phosphorylation by impeding the ROCK1-PTEN signaling pathway.	5xFAD mice/SH-SY5Y	Temporal cortex	Blood-derived exosomes	[121]
Neuroinflammation	let-7-a/e	Activated microglia cells and enhanced inflammatory cytokine production.	Murine BV2 microglial cells		Blood-derived exosomes	[123,128]
	let-7b/e	Enhanced inflammatory responses by acting as extracellular signaling molecules and as ligands for TLR7 in neuronal cells.	C57Bl/6J mice/RAW264.7, N1E-115, and HEK293 cells		CSF-derived exosomes	[125,126]
	miR-125b	Enhanced inflammatory responses by promoting pro-inflammatory cytokine production (NF-α, IL-1β, and IL-6).	Hippocampal and cortical neurons of rat/neuroblastoma Neuro2a APPSwe/Δ9 cells		CFS- and blood-derived exosomes	[92,110,129]
	miR-146a	Promoted microglial shifting towards the M2 phenotype and reduced pro-inflammatory cytokines production.	C57BL/6J and APP/PS1mice/HMC3 and SH-SY5Y cells		Blood-derived exosomes	[132,134]
		Attenuated astrocytic inflammation by inhibiting NF-κB activation.	APP/PS1 mice/BM-MSC cells seeded with astrocytes			[133,134]
	miR-132	Enhanced the expression of VE-cadherin junction and improved BBB integrity.	Primary cortical neurons cells	Temporal cortex, prefrontal cortex, and hippocampus	Blood-derived exosomes	[117,137]
Synaptic Dysfunction	miR-146a	Downregulated the expression of presynaptic *SYT1* and postsynaptic *Nlg1* genes.	3xTg-AD mice/primary cortical neurons	Temporal cortex, frontal cortex, and cerebellum		[38,134]
	miR-34a	Downregulated the expression of key synaptic genes including *NR2A*, *VAMP2*, and *SYT1*.	Mixed glial and neuron cell cultures		Blood-derived exosomes	[39]
	miR-30	Repressed synaptic genes including sirt1, *ephB2*, and *GluA2*.	5XFAD APP transgenic mice/mixed neurons and astroglial cells	Hippocampus		[145]

## 5. Exosomal miRNAs as Promising Diagnostic Biomarkers for AD

Early AD diagnosis is crucial, given the long preclinical period and the need for effective treatment strategies. Identifying reliable biomarkers to detect neurological alterations in the brain is essential for developing targeted therapeutic approaches. As the significant regulatory functions of exosomal miRNAs in AD, combined with acting as a mediator in intercellular communication, exosomal miRNAs provide great potential to enhance diagnostic accuracy and serve as specific biomarkers for AD. Several studies have investigated these miRNAs in exosomes derived from blood and CSF of AD patients, as shown in Table 2.

### 5.1. Exosomal miRNA Dysregulation in CSF

Exosomal miRNAs derived from CSF are considered promising methods for identifying prognostic and diagnostic biomarkers for AD. Gui et al. investigated the possibility of utilizing exosomal miRNAs as AD diagnostic biomarkers by analyzing their differential expression in CSF of AD patients. The results revealed differential expression of miR-132-5p, miR-16-2, miR-29c, miR-136-3p, miR-331-5p, and miR-485-5p in patients with AD compared to healthy individuals [80]. Another study observed that the levels of two particular miRNAs, miR-9 and miR-598, were highly expressed in the exosomes derived from CSF samples of AD patients compared to those in control individual’s CSF samples. Importantly, these miRNAs were more abundant in CSF-derived exosomes than in the whole CSF, specifically in the samples obtained from AD patients [146], indicating a selective encapsulation and enrichment of these particular miRNAs within extracellular exosomes. Other studies have found that miR-193b and miR-135a expression is higher in CSF-derived exosomes of MCI and AD patients than in healthy individuals [147,148]. McKeever and colleagues compared the exosomal miRNA expression profiles derived from CSF patients with YOAD and LOAD. The results revealed alterations in miR-605-5p, miR-451a, and miR-125b-5p levels in the exosomes derived from CSF of both AD groups. Interestingly, dysregulation of exosomal miR-16-5p was specific to the YOAD group but not the LOAD group, indicating the potential for using it as a particular biomarker associated with the pathological processes underlying the earlier onset of AD [92].

Exosomal miRNAs isolated from CSF may also serve as promising biomarkers for distinguishing AD from other types of dementia. Tan and colleagues investigated the potential of using exosomal miRNAs isolated from CSF as biomarkers to distinguish AD from frontotemporal dementia (FTD). The results revealed a significant reduction in two exosomal miRNAs, miR- 320a and miR-204-5p, in the CSF samples of both dementia groups compared to healthy individuals. Additionally, they observed that exosomal miR-328-3p exhibited significant dysregulation in AD patients but not in the FTD patient group and demonstrated moderate diagnostic value with an area under the curve (AUC) of 0.702. Importantly, exosomal miR-328- 3p significantly correlated with the reduction in Aβ42 in the CSF, suggesting that it is potentially utilized as a disease-specific biomarker to help differentiate AD from FTD [78].

### 5.2. Exosomal miRNA Dysregulation in the Blood

Given the challenges and constraints of obtaining CSF samples, growing interest has been in identifying exosomal miRNA biomarkers from peripheral blood. Investigators have examined different blood components, including plasma or serum, to search for reliable biomarkers of AD that demonstrate high sensitivity and specificity for accurate diagnosis. Lugli and colleagues investigated the possibility of using miRNAs as AD diagnostic biomarkers by analyzing their differential expression in plasma exosomes from AD patients. The analysis results indicated that a specific group of seven miRNAs could reliably predict AD status, achieving an accuracy ranging from 83% to 89%. Among these miRNAs, miR-342-3p appeared to be particularly significant, showing decreased expression in AD patients and exhibiting a robust association with several other downregulated miRNAs in AD [107]. Another study revealed dysregulation in the expression of exosomal Let-7g-5p, miR-126-3p, miR-142-3p, miR-146a-5p, and miR-223-3p in AD patients, and they showed strong correlations with disease severity [134]. Nie et al. discovered that eight miRNAs obtained from plasma exosomes displayed significant dysregulation in AD patients. Among these miRNAs, Let-7e-5p, miR-125a-5p, miR-23a-3p, and miR-375 were determined as AD diagnostic biomarkers in separate investigations examining various blood components [150,151,152,153,154]. This consistency suggests these miRNAs could serve as reliable AD biomarkers across different blood-based assessments. Additionally, research has identified significant dysregulation in the expression of six miRNAs in the blood-derived neurogenic exosomes of AD patients: miR-23a-3p, miR-223-3p, and miR-190a-5p were upregulated, while miR-132 and miR-100-3p were downregulated [139,155]. These results suggest that assessing the expression of these miRNAs in neurogenic exosomes could complement existing approaches for diagnosing AD. Cheng et al. examined exosomal miRNA profiles in the serum of individuals with AD, and they identified a set of 16 miRNA biomarkers. Importantly, when this set of exosomal miRNAs was incorporated with established risk agents for AD, such as sex, age, and APOE4 ε4 genotype status, the combined method achieved a high prediction accuracy of AD with 87% sensitivity and 77% specificity. These results indicate that integrating the analysis of exosomal miRNAs with other risk agents could improve the accuracy and effectiveness of blood-based tests for the early detection and diagnosis of AD [156]. Another study found a major reduction in exosomal miR-223 levels in the blood samples of AD patients, and its reduction was strongly associated with both the severity of cognitive decline and neuroinflammation [157], indicating its potential use as a biomarker for measuring and monitoring these processes related to AD. A recent investigation detected a significant downregulation in the expression of three miRNAs (miR-30b-5p, miR-22-3p, and miR-378a-3p) in serum-derived exosomes from AD patients. Combining these three exosomal miRNAs achieved a high level of accuracy in AD diagnosis with an AUC value of 0.88 [158].

Exosomal miRNAs isolated from blood may also serve as promising biomarkers for differentiating AD from other types of dementia. Gámez-Valero et al. investigated the potential of using exosomal miRNAs derived from the blood to differentiate between AD and dementia with Lewy bodies (DLB). After analyzing exosomal miRNA differential expression, they revealed that four exosomal miRNAs significantly reduced their expression in AD patients compared to healthy individuals. Interestingly, two specific miRNAs, miRNA-21-5p and miR-451, showed significant downregulation in AD patients in comparison to those with DLB. These two miRNAs demonstrated high differentiation power, with AUC values of 0.9, effectively distinguishing between AD and DLB [159]. In a comprehensive comparative study, researchers analyzed exosomal miRNA expression profiles in different neurodegenerative disorders, including AD, Parkinson’s disease (PD), vascular dementia (VD), and vascular parkinsonism (VP). One of the interesting results was the alteration in the expression of miR-23a in all disease groups, suggesting its potential as a biomarker for neurodegenerative conditions. Moreover, miR-34b, miR-125b, and miR-130b showed specific changes in Alzheimer’s-like disorders, indicating that these particular neuro-miRNAs may contribute to AD pathogenesis and could serve as disease-specific biomarkers [110]. Liu and colleagues thoroughly analyzed the differential expression of exosomal miRNAs isolated from blood samples across multiple neurodegenerative conditions, including AD, MCI, and VD. Their analysis demonstrated that a set of 18 miRNAs was differentially expressed in the exosomes of AD patients compared to healthy individuals. Among these, miR-93-5p, miR-424-5p, miR-1306-5p, and miR-3065-5p exhibited distinct expression patterns in samples from AD patients compared to those from patients with MCI and VD [160].

**Table 2:** Exosomal miRNA signatures with clinical diagnostic potential for AD.

Source	Study Populations	RNA Identification	Downregulated	Upregulated	Efficiency of Diagnosis	Ref.
CSF	PD [*n* = 47], AD [*n* = 28], and Ctrl [*n* = 27]	Microarray and qRT-PCR	miR-16-2 miR-29c miR-136-3p miR-331-5p	miR-132-5p miR-485-5p	-	[80]
	AD [*n* = 10] and Ctrl [*n* = 10]	Microarray and qRT-PCR	miR-598 miR-9-5p		-	[146]
	LOAD [*n* = 13], EOAD [*n* = 17], and Ctrl [*n* = 12]	Microarray and qRT PCR	miR-16-5p miR-451a miR-605-5p	miR-125b-5p	-	[92]
	AD [*n* = 28], FTD [*n* = 12], and Ctrl [*n* = 8]	qRT PCR	miR-320a miR-328-3p miR-204-5p	-	AUC 0.702	[78]
CSF and Blood (Serum)	AD [*n* = 51], MCI [*n* = 43], and Ctrl [*n*= 84]	qRT-PCR	miR-193b	-	-	[89]
	SCD [*n* = 89], MCI [*n* = 92], AD [*n* = 92], and Ctrl [*n* = 60]	qRT-PCR	-	miR-193b	-	[147]
	SCD [*n* = 165], MCI [*n* = 143], DAT [*n* = 202], and Ctrl [*n* = 30]	qRT-PCR	-	miR-135a	-	[148]
Blood (Plasma)	AD [*n* = 35] and Ctrl [*n*= 35]	NGS	miR-23b-3p miR-141-3p miR-185-5p miR-342-3p miR-342-5p miR-338-3p miR-3613-3p	-	AUC: 0.91 Accuracy between 83 and 89%	[107]
	DLB [*n* = 18], AD [*n* = 10], and Ctrl [*n* = 15]	NGS and qRT-PCR	miR-451a miR-21-5p miR-23a-3p miR-126-3p let-7i-5p miR-151a-3p	-	AUC: 0.9	[159]
	AD [*n* = 16], MCI [*n* = 16], and Ctrl [*n*= 31]	Microarray and qRT-PCR	miR-132-3p miR-212	-	AUC: 0.55–0.84	[139]
	AD [*n* = 40] and Ctrl [*n* = 40]	NGS	miR-100-3p	miR-23a-3p miR-223-3p miR-190-5p	-	[155]
	AD [*n* = 5], PD [*n* = 7], and Ctrl [*n* = 34]	NGS	miR-204-5p miR-125a-5p miR-1468-5p miR-375 let-7e-5p	miR-423-5p miR-369-5p miR-23a-3p	-	[150]
	AD [*n* = 42] and Ctrl [*n* = 19]	NanoString and qRT-PCR	let-7g-5p miR126-3p miR142-3p miR-146a-5p mir223-3p	-	-	[135]
Blood (Serum)	AD [*n* = 23], MCI [*n* = 3], and Ctrl [*n*= 23]	NGS and qRT-PCR	miR-15b-3p miR-342-3p miR-1306-5p	miR-15a-5p miR-18b-5p miR-20a-5p miR-30e-5p miR-93-5p miR-101-3p miR-106a-5p miR-106b-5p miR-143-3p miR-335-5p miR-361-5p miR-424-5p miR-582-5p miR-3065-5p	Sensitivity [90] and specificity [77%]	[156]
	AD [*n* = 22], VD [*n* = 10], and Cotrl [*n* = 16]	qRT-PCR	miR-223	-	AUC 0.875	[157]
	AD [*n* = 30], PD [*n* = 30], VD [*n* = 24], VP [*n* = 25], and Ctrl [*n* = 30]	qRT-PCR	miR-34b miR-130b	miR-23a miR-125b	AUC 0.83–0.92	[137]
	AD [*n* = 31], MCI [*n* = 10], VD [*n* = 10], and Ctrl [*n* = 10].	qRT-PCR	miR-1306-5p miR-342-3p miR-15b-3p	miR-93-5p miR-424-5p miR-3065-5p	-	[160]
	AD [*n* = 8] and Ctrl [*n* = 8]	NGS and qRT-PCR	miR-30b-5p miR-22-3p miR-378a-3p	-	AUC 0.668, 0.637, and 0.718	[158]

## 6. Exosomal miRNAs with Therapeutic Potential in AD

Despite being the most prevalent neurodegenerative disorder, AD still lacks effective therapies that can halt or reverse its progression. Current drug interventions, including acetylcholinesterase inhibitors (e.g., donepezil and rivastigmine) and the NMDA receptor antagonist (memantine), primarily provide improvements in cognitive symptoms but do not address the underlying disease mechanisms [161]. Recent advances in disease-modifying therapies aim to target hallmark AD pathologies, such as amyloid-beta plaques, tau aggregation, and neuroinflammation [162]. However, these therapies often exhibit limited efficacy, particularly in the later stages of the disease, and are largely focused on a single pathological target. Given the multifactorial nature of AD, a more integrative, multi-target therapeutic approach may be required for more effective intervention.

In this context, exosomal miRNAs present a promising new therapeutic avenue. Exosomal miRNAs are protected from degradation and have the ability to cross the BBB, allowing for targeted delivery to the CNS [160]. These miRNAs can modulate several genes involved in the core pathological mechanisms of AD, offering the potential to simultaneously address various aspects of the disease [163]. This dual-action approach not only holds promise for slowing disease progression but also offers the possibility of personalized treatments based on an individual’s unique miRNA profile. As a result, this innovative therapeutic strategy could pave the way for more effective, comprehensive treatments that address the complex and diverse nature of AD.

Studies using the AD animal model and in vitro have confirmed that exosomes effectively deliver miRNAs to target cells. This delivery mechanism has the potential to restore normal gene expression patterns in these cells, thereby alleviating underlying disease symptoms and potentially slowing or even reversing AD progression, as highlighted in Table 3. One experimental study has shown that mesenchymal stem cell (MSC)-derived exosomes delivering miR-223 to neurons reduced neural cell death by targeting the PTEN-PI3K/Akt signaling pathway, which plays a crucial role in cell survival and neuroprotection [164]. Another study demonstrated that exosomes derived from microglia effectively transport miR-124-3p to hippocampal neurons, where it targets Rela, an inhibitory transcription factor of ApoE, resulting in the prevention of β-amyloid deposition and an improvement in cognitive function in mouse models [165].

Additionally, delivering miR-124-3p to astrocytes induces their differentiation and upregulates the expression of glutamate transporters, protecting against glutamate excitotoxicity and neuronal death [166]. A separate study revealed that transplanting exosomes loaded with miR-29 into hippocampal neurons significantly alleviated cognitive dysfunction induced by Aβ neurotoxicity in rats with AD. This beneficial effect was further investigated in vitro experiments, which demonstrated that delivering exosomal miR-29 to neuronal cells led to the downregulation of *BACE1* mRNA expression, ultimately reducing Aβ accumulation and mitigating neurodegeneration [167]. Nakano and colleagues found that administering exosomes derived from bone marrow mesenchymal stem cells (BM-MSCs), packed with miR-146a, led to improvements in cognitive function in mice with AD. This therapeutic effect was driven by the uptake of these exosomes into astrocytes, where miR-146a suppressed NF-κB mRNA expression. This, in turn, alleviated astrocyte-mediated inflammation and promoted the formation of synapses in the hippocampus, contributing to improved cognitive outcomes [133]. Similarly, administering exosomal miR-146a derived from BM-MSCs alleviated cognitive decline induced by diabetes via reducing astrocyte-mediated inflammation in mice [168]. In a dementia mouse model, systemic administration of MSC-derived exosomes enriched with miR-132-3p promoted the recovery of cognitive function by improving both neuronal and synaptic dysfunction. This therapeutic effect was mediated through the activation of the Ras/Akt/GSK-3β pathway, which plays a vital role in tau metabolism and synaptic plasticity. Additionally, miR-132-3p enriched exosomes decreased Aβ levels in the cortex and hippocampus of dementia mice [169].

**Table 3:** Exosomal miRNAs as potential targets of AD treatment.

miRNA	Cell Source	Recipient Cell	Target Gene/Pathway	Potential Mechanisms in AD Treatment	Ref.
miR-223	MSCs	Neurons	PTEN-PI3K/Akt pathway	Decreases neural cell death, promoting neuroprotection and cell survival	[164]
miR-124-3p	Microglia	Neurons	Rela/ApoE pathway	Alleviates neurodegeneration induced by Aβ cytotoxicity and improves cognitive functions	[165]
	Neurons	Astrocytes	p38 MAPK/GLT-1 pathway	Enhances glutamate uptake, reducing excitotoxicity and protecting neuronal cells from damage	[166]
miR-29	MSCs	Neurons	*BACE1* mRNA	Improves cognition dysfunction induced by Aβ neurotoxicity by downregulation of *BACE1* mRNA	[167]
miR-146a	BMSCs	Astrocytes	*NF-κB* mRNA	Reduces astrocyte-mediated inflammation, improving synaptic formation and cognitive function	[133,168]
miR-132	MSCs	Neurons	Ras/Akt/GSK-3β pathway	Enhanced recovery cognitive functions by improving synaptic and neuronal dysfunction	[169]

## 7. Conclusions and Future Directions

Exosomes play an essential role in facilitating neuronal communication, with miRNAs serving as mediators within the exosome-mediated cell communication network. The collaborative or independent actions of exosomes and miRNAs contribute to regulating the homeostasis of the brain microenvironment. Glial and neuronal cells secrete exosomes carrying miRNAs, and any dysregulation in those miRNAs can significantly impact the pathogenesis of AD. Importantly, detecting changes in exosomal miRNA profiles in patient biofluids, even during the earliest stages, holds promise as a valuable strategy for the earliest AD diagnosis. Additionally, the ability of exosomes to cross the BBB allows for the loading of exogenous miRNAs into exosomes, facilitating targeted delivery to affected regions. This represents an innovative advancement in the treatment of AD.

Although the strategies and potential of exosomal miRNA research in AD are promising, this field is still in its early stages, and further investigations are required. There are significant gaps in understanding the disease mechanisms, identifying AD-specific exosomal miRNAs, and unravelling the complex miRNA network. These miRNAs have diverse functions, exhibit region-specific expression in the brain, and contribute to AD through complex pathways at various stages of the disease. Therefore, addressing these challenges is crucial for advancing our understanding of AD pathogenesis. Additionally, comprehensive studies involving diverse cohorts, longitudinal assessments, and rigorous validation are necessary to identify the key mechanisms underlying miRNA-AD interactions. This will offer valuable insights into the molecular mechanisms associated with AD, facilitate the identification of reliable biomarkers, and potentially aid in the development of therapies to enhance cognitive function in individuals with AD.

## Data Availability

Data will be made available on request.
